# Clinical value of the water injection, dilution, and drainage method for blind nasoenteric tube placement in patients receiving sequential enteral nutrition support after major thoracic surgery

**DOI:** 10.3389/fonc.2026.1838370

**Published:** 2026-05-19

**Authors:** Kunkun Cheng, Le Zhang

**Affiliations:** Outpatient Nursing Group, Jiangsu Cancer Hospital & Jiangsu Institute of Cancer Research & The Affiliated Cancer Hospital of Nanjing Medical University, Nanjing, China

**Keywords:** blind nasoenteric tube insertion, nursing, sequential enteral nutrition support, severe thoracic surgery, water injection, dilution and drainage method

## Abstract

**Aim:**

This study aimed to evaluate the clinical effectiveness of a blind nasoenteric tube placement technique based on water injection, dilution, and drainage in patients receiving sequential enteral nutrition support after major thoracic surgery.

**Methods:**

Eighty patients who underwent major thoracic surgery were enrolled and equally assigned to an observation group and a control group. The control group received conventional blind nasoenteric tube placement using gas injection, whereas the observation group received the water injection, dilution, and drainage method. The evaluated outcomes included catheterization success rate, catheterization time, length of hospital stay, nutritional indicators, serum nutritional markers, inflammatory response markers, immune function markers, quality of life, and incidence of adverse events.

**Results:**

Compared with the control group, the observation group showed significantly shorter catheterization time and hospital stay, as well as a significantly higher catheterization success rate (P < 0.05). After the intervention, both groups demonstrated improvements in body mass index (BMI), triceps skinfold thickness (TSF), mid-arm muscle circumference (MAMC), hemoglobin, serum albumin, transferrin levels, and quality of life scores. These improvements were more pronounced in the observation group than in the control group (P < 0.05). In addition, after the intervention, the observation group had significantly lower levels of inflammatory markers, including C-reactive protein (CRP), procalcitonin (PCT), and interleukin-6 (IL-6), and significantly higher levels of immune function markers, including CD3^+^, CD4^+^, and the CD4^+^/CD8^+^ ratio, compared with the control group (P < 0.05). Moreover, the incidence of adverse reactions was significantly lower in the observation group (P < 0.05).

**Conclusion:**

The blind nasoenteric tube placement protocol based on water injection, dilution, and drainage is associated with improved catheterization success and procedural efficiency in patients receiving sequential enteral nutrition support after major thoracic surgery. The observed improvements in nutritional status, inflammatory markers, immune function, and quality of life are likely indirect and should not be attributed to the catheterization technique itself. Given the presence of multiple co-interventions and the lack of intervention isolation, these findings should be interpreted as associative and hypothesis-generating rather than causal.

## Introduction

Patients after severe thoracic surgery (including major procedures such as lung resection, esophageal surgery, and mediastinal tumor resection) are in a state of stress due to the disease itself and the surgical intervention, with energy metabolism exceeding the physiological resting level by up to 50% (1). Furthermore, abnormal swallowing function can lead to insufficient nutrient intake, resulting in malnutrition, which adversely affects prognosis and quality of life (2). Consequently, implementing timely and scientific nutritional support measures is of great significance for these patients.

In nutritional support, “sequential therapy” encompasses two main concepts (3). First, it refers to sequential nutritional support, transitioning from early combined enteral and parenteral nutrition to total enteral nutrition, emphasizing the gradual shift from combined use to exclusive enteral nutrition (4). Second, it involves sequential early enteral nutrition support, which starts with early enteral nutrition based on amino acids or peptides and progresses to a total protein-based formula, with daily increments in infusion volume and rate (5).

For enteral nutrition in patients after severe thoracic surgery, retropyloric feeding is prioritized as it can effectively decrease the incidence of aspiration pneumonia and improve patient outcomes. The placement of a nasoenteric tube is a prerequisite for this (6). Although the self-propelled spiral nasointestinal tube – which relies on its tip’s propulsion and gastrointestinal motility - is currently used in clinical practice for its simplicity and cost-effectiveness, its success rate is often low due to various factors (7).

Our study was designed to explore the application effect of blind nasoenteric tube insertion by water injection, dilution, and drainage method in patients undergoing sequential enteral nutrition support therapy after severe thoracic surgery.

## Data and methods

### General data

Eighty patients who had undergone severe thoracic surgery (defined in this study as major thoracic procedures including lung resection, esophageal surgery, and mediastinal surgery requiring postoperative intensive monitoring and nutritional support) were selected as study participants. Inclusion criteria: (1) Critically ill patients in the department of thoracic surgery; (2) Age >18 years old; (3) Patients and their families gave informed consent to this study; (4) Patients with impaired gastric motility, gastroparesis and intolerance to gastric feeding. Exclusion criteria: (1) Patients with esophageal hemorrhage, esophageal varicose veins, intestinal obstruction, intestinal paralysis, intestinal perforation, intestinal necrosis and other acute abdominal diseases; (2) Patients with abnormal liver and kidney function or malignant tumor; (3) Patients with mental and behavioral disorders or serious primary cerebrovascular diseases.

### Sample size calculation

*A priori* sample size calculation was implemented using G*Power software (version 3.1.9.7) based on the primary outcome of catheterization success rate. Assuming a two-tailed test with α = 0.05, power (1-β) = 0.80, and a large effect size (w = 0.5) based on preliminary experiments, the estimated required sample size was 34 patients per group. To account for potential dropouts, 40 patients per group (total 80 patients) were enrolled.

### Randomization, allocation concealment and blinding

Randomization was performed by means of a computer-generated random number table with a 1:1 allocation ratio. Allocation concealment was ensured by sequentially numbered, opaque, sealed envelopes, which were opened only after patient enrollment and immediately prior to intervention. Due to the nature of the intervention (different catheterization techniques), blinding of the operators was not feasible. However, outcome assessors (radiologists interpreting X-ray films and laboratory personnel analyzing blood samples, and flow cytometry technicians analyzing immune markers) were blinded to group allocation to minimize detection bias.

### Operator training and procedure standardization

All catheterization procedures in both groups were performed by two experienced attending physicians from the Department of Critical Care Medicine, each with over 5 years of clinical experience in blind nasoenteric tube placement. Prior to the study initiation, both operators underwent a standardized training program consisting of didactic instruction on both techniques, supervised practice on mannequins, and observation of 5 procedures for each technique performed by an expert gastroenterologist. Inter-operator reliability was assessed by having both physicians independently perform the procedure on 5 pilot patients (not included in the final analysis), with 100% agreement on catheterization success and positioning confirmation. To minimize potential performance bias, each operator performed an approximately equal number of procedures in both groups, and no significant differences in outcomes were observed between operators in preliminary analyses.

### Treatments

Forty-eight hours after conventional treatment, sequential enteral nutrition therapy was initiated. A nasoenteric tube was inserted, and an enteral nutrition suspension (SP) (produced by Nutricia Pharmaceutical (Wuxi) Co., LTD.) was used within 1–3 days of treatment. After being heated to 35 °C, SP was administered through the nasoenteric tube at 50–100 mL per administration, once every 2–3 hours. If the patient had no adverse reactions such as abdominal distension or diarrhea during treatment, the dosage of suspension was increased to 150–180 ml/time. On the first day of injection, the volume was limited to 500 ml, and then the dosage gradually increased to 84–126 kJ/(kg·d). Starting from the 4th day, the patient was given enteral nutrition suspension (TPF) (produced by Nudidia Pharmaceutical (Wuxi) Co., LTD.) with the same usage and dosage as the 3rd day, continuous treatment for 2 weeks.

## Methods

Control group: Enteral nutrition was administered by blind nasoenteric tube insertion using gas injection. The specific steps were as follows: preparing the necessary items before catheterization, instructing patients to fast for 4–6 hours, administering a prokinetic agent by intramuscular injection 15 to 30 minutes before catheterization, and placing the patients in a low semi-decumbent position. Then, the nasogastric tube was placed in the stomach as the first marker according to the standard nasogastric tube insertion procedure (according to the measured length). After judging the gastric cavity, the pH of gastric fluid was extracted for testing or the sound of air passing water was heard, and 5–10 ml/kg of gas was injected into the stomach. 5 min later, the patient was instructed to turn to the right lateral position and slowly place the tube at 75 cm, which was usually marked as the second place (pylorus to duodenum). After determining the location, the catheter was pumped back, the pH value of the pumped fluid was monitored, and it was determined by stethoscopy that another 90 cm was inserted into the stomach to reach the jejunum position, and then X was taken of the abdomen wire to determine the position, and the guide wire was withdrawn and fixed.

Observation group: Enteral nutrition was administered by blind nasoenteric tube insertion by water injection, dilution and drainage method. The key procedural steps are illustrated in **Supplementary Figure 1**. 500 ml of warm water was added to the preparation of items before catheterization, the patient was instructed to refrain from eating for more than 6 h, and intravenous metoclopramide was injected 3–5 minutes before catheterization to promote gastric motility. The patient was instructed to take a supine or semi-seated position, and then the nasogastric tube was advanced to the first marker to the first marker in the stomach (according to the measured length), minus 3–5 cm, and the gastric injection method was used to inject water at 5–10 ml/kg. The patient did not need to change the body position, and after the catheter slowly advanced to 75 cm, a 20 ml syringe was used to withdraw the negative pressure. If the negative pressure clearly indicated that the catheter might be in the intestine, the intestinal fluid was repeatedly extracted three times; if no intestinal fluid was extracted, auscultation was given; if the auscultation and negative pressure were both obvious, the next step was taken. A 20 ml syringe was used to pump 15 ml of warm water into the catheter. If less than 5 ml of warm water was pumped back, the above method was repeated for aspiration, and the catheter was rotated at the same time, repeatedly aspirated back into the syringe to dilute the liquid for pH test paper. After extracting the intestinal fluid, the tube was slowly placed until 90 cm, and then the intestinal fluid was extracted again without stethoscopy. The abdomen was then X-rayed to determine the position, the guide wire was withdrawn and fixed. If catheterization failure occurred, the tube was withdrawn to 50–55 cm (in the stomach) at the first failure, and the catheterization path was changed after repeated failure. If a tube was placed under a gastroscope, the spiral naso-intestinal tube passively waiting to pass through the pylorus was a remedy for direct implantation failure, specifically: (1) The guide wire was measured at 75 cm (near the gastric pylorus), and 25–40 cm was reserved for fixing in the earlobe or cheek, and a scale was marked on the tube wall to prevent the guide wire from moving; (2) With the gastric peristaltic catheter passing through the pylorus on its own occurring within 24 h, the patient may be placed in a semi-sloped position and turned to the right to accelerate passage through the pylorus; (3) Patients were given gastric motile drugs such as metoclopramide hydrochloride, rhubarb, erythromycin or water injection. Importantly, in addition to the catheterization technique itself, the two groups differed in several procedural elements, including the administration route and timing of prokinetic agents, patient positioning, and specific workflow steps. These differences reflect real-world clinical protocols but indicate that the intervention represents a combined procedural strategy rather than a strictly isolated technical comparison.

### Primary outcome

The primary outcome of this study was the catheterization success rate, defined as correct positioning of the tube tip beyond the pylorus (in the duodenum or jejunum) confirmed by abdominal X-ray, interpreted by a radiologist blinded to group allocation. Catheterization time was measured from the moment of nasal insertion to final X-ray confirmation.

### Secondary outcomes

All other outcomes were considered secondary or exploratory outcomes, including catheterization time, hospital stay, nutritional indicators, serum nutritional markers, inflammatory and immune markers, quality of life, and adverse events.

Nutritional indicators, which contained body mass index (BMI), triceps skin fold thickness (TSF) along with arm muscle circumference (MAMC), were compared.

Nutritional serum indexes including hemoglobin, serum albumin, and transferrin were compared.

Inflammatory and immune markers: Peripheral venous blood samples were collected from all patients before the intervention and after 2 weeks of treatment. Inflammatory markers, including C-reactive protein (CRP), procalcitonin (PCT), along with interleukin-6 (IL-6), were measured using standard immunoassay techniques. Cellular immune function markers, including CD3^+^, CD4^+^ T-lymphocyte percentages and the CD4^+^/CD8^+^ ratio, were determined using flow cytometry.

Generic quality of life inventory-74 (GQOLI-74) was adopted to assess the quality of life (8). The scale included material life, physical function, psychological function along with social function, involving 74 items, each item scored 1–5 points, and the total score was 74-370. A higher score represented a better quality of life for the patient.

The occurrence of adverse events including aspiration, pulmonary infection, upper gastrointestinal hemorrhage, vomiting, diarrhea, and abdominal distension was compared. Adverse events were prospectively recorded daily by nurses who were blinded to group allocation, using pre-defined criteria: aspiration (respiratory symptoms with witnessed regurgitation or tube feeding detected in tracheal secretions), pulmonary infection (new or progressive infiltrate on chest X-ray plus at least two of: fever >38 °C, leukocytosis, or purulent secretions), vomiting (expulsion of gastric contents), diarrhea (>3 loose stools per day), and abdominal distension (patient-reported discomfort with visible abdominal distention).

### Statistical analysis

All statistical analyses were performed using SPSS software (version 23.0, IBM Corp., Armonk, NY, USA). Continuous variables were tested for normality using the Shapiro-Wilk test and are presented as mean ± standard deviation (x ± s). Categorical variables are presented as frequencies and percentages.

For primary analyses, between-group comparisons of continuous variables (e.g., catheterization time, hospital stay, nutritional parameters, inflammatory/immune markers, quality of life scores) were conducted using independent-sample t-tests. Within-group comparisons of pre- and post-intervention measurements were performed using paired t-tests. Categorical variables (e.g., catheterization success rate, adverse events) were compared between groups using the χ² test or Fisher’s exact test when expected cell counts were less than 5.

To address potential confounding and strengthen causal inference, multivariable analyses were performed. For continuous outcomes (e.g., nutritional parameters, quality of life scores), analysis of covariance (ANCOVA) was conducted with group assignment as the fixed factor and baseline values of the respective outcome measures as covariates. This approach adjusts for any baseline differences and provides an estimate of the treatment effect independent of initial values. For binary outcomes (e.g., catheterization success), multivariable logistic regression was performed, adjusting for potential confounders including age, sex, surgical type, baseline APACHE II score, and baseline nutritional status (BMI and serum albumin). Adjusted odds ratios (OR) with 95% confidence intervals (CI) were calculated.

All analyses were conducted using both per-protocol and intention-to-treat principles. Given that there were no protocol violations, losses to follow-up, or missing data, both approaches yielded identical results. Statistical significance was set at P < 0.05 (two-tailed). To account for multiple comparisons across different outcomes, Bonferroni correction was applied where appropriate, with adjusted significance thresholds indicated in the corresponding figure legends.

## Results

### Baseline data between the two groups

A total of 96 patients were assessed for eligibility. Of these, 16 were excluded prior to randomization (10 did not meet inclusion criteria, 4 declined to participate, and 2 were excluded for other reasons). The remaining 80 patients were randomized equally to the observation group (n = 40) and control group (n = 40). All 80 patients received their allocated intervention, and no patients were lost to follow-up or discontinued the intervention during the 2-week study period. Therefore, data from all 80 randomized patients were included in the final analysis, and both per-protocol and intention-to-treat analyses were conducted, yielding identical results due to the absence of protocol violations or dropouts.

Baseline clinical characteristics, including age, sex, surgical type, disease severity assessed by the Acute Physiology and Chronic Health Evaluation II (APACHE II) score, and baseline nutritional status (BMI and serum albumin), were compared between the two groups (**Table 1**). No significant differences were observed in baseline characteristics between the two groups (P > 0.05).

**Table 1:** Baseline data between the two groups.

Characteristic	Observation group (n=40)	Control group (n=40)	t/χ^2^	P
Age (years)	50.32 ± 10.68	50.38 ± 10.74	0.025	0.980
Sex			0.200	0.654
Male	20 (50.00)	22 (55.00)		
Female	20 (50.00)	18 (45.00)		
Surgical type			0.068	0.966
Lung resection	18 (45.00)	17 (42.50)		
Esophageal surgery	12 (30.00)	13 (32.50)		
Mediastinal surgery	10 (25.00)	10 (25.00)		
APACHE II score	15.42 ± 3.21	15.58 ± 3.35	0.218	0.827
BMI (kg/m²)	22.15 ± 2.43	22.28 ± 2.51	0.235	0.814
Serum albumin (g/L)	34.26 ± 3.52	34.41 ± 3.48	0.191	0.848

### The catheterization time, catheterization success and hospital stay in 2 groups

**Figure 1:**
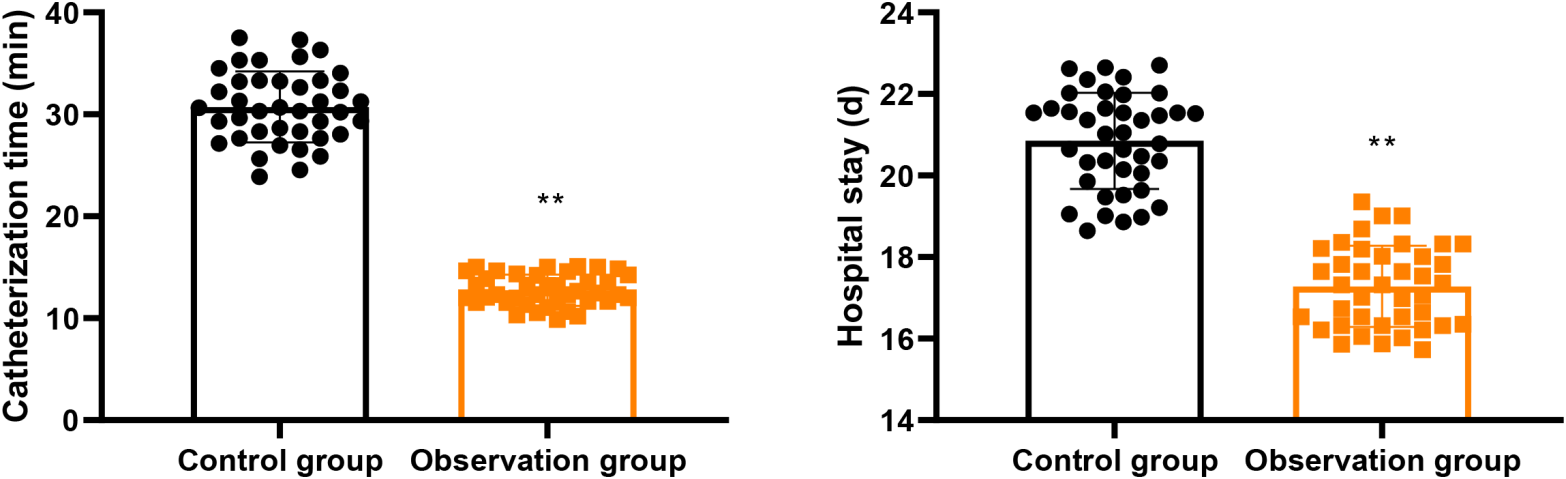
Catheterization time and hospital stay in 2 groups. ^**^P<0.01.

In contrast to the control group, the catheterization time as well as hospital stay in the observation group were lower (P < 0.01, **Figure 1**). Meanwhile, the cases of catheterization success in the observation group were higher compared with the control group (P < 0.05, **Table 2**). After adjusting for potential confounders (age, sex, surgical type, APACHE II score, and baseline nutritional status) using multivariable logistic regression, the observation group remained independently associated with higher catheterization success rate (adjusted OR = 3.42, 95% CI: 1.38-8.47, P = 0.008). Similarly, the reduction in catheterization time and hospital stay remained significant after adjustment in ANCOVA models (P < 0.01 for both).

**Table 2:** Catheterization success in 2 groups.

Groups	n	Cases of catheterization success	Percentage of catheterization success
Control group	40	30	75.00%
Observation group	40	38	95.00%
χ^2^		6.275	
P		0.012	

### BMI, TSF and MAMC in 2 groups

**Figure 2:**
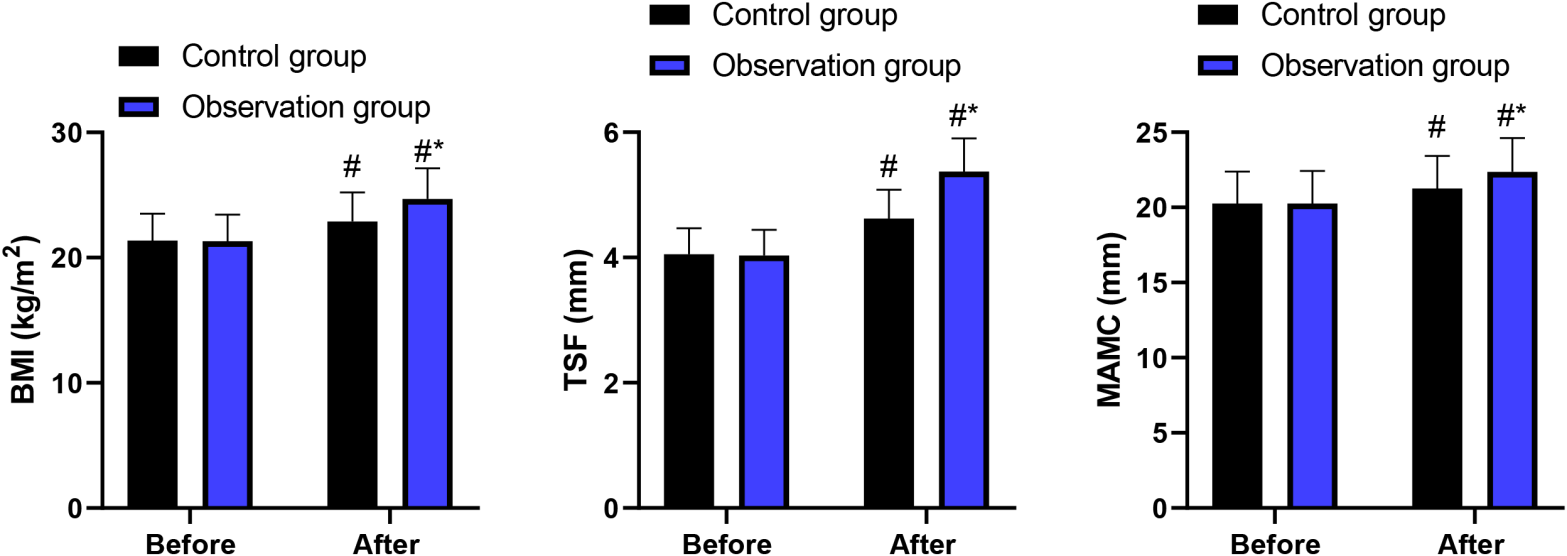
BMI, TSF and MAMC in 2 groups. ^#^P<0.05, in contrast to before intervention. ^*^P<0.05, in contrast to control group.

Before intervention, no significant difference was observed in BMI, TSF and MAMC between 2 groups (P > 0.05). After intervention, BMI, TSF and MAMC were increased in 2 groups, and those in the observation group presented higher when comparing with the control group (P < 0.05, **Figure 2**). ANCOVA analyses adjusting for baseline values confirmed that the observation group had significantly greater improvements in BMI (F = 6.84, P = 0.011), TSF (F = 5.92, P = 0.017), and MAMC (F = 7.23, P = 0.009) compared to the control group.

### Hemoglobin, serum albumin, and transferrin levels in 2 groups

**Figure 3:**
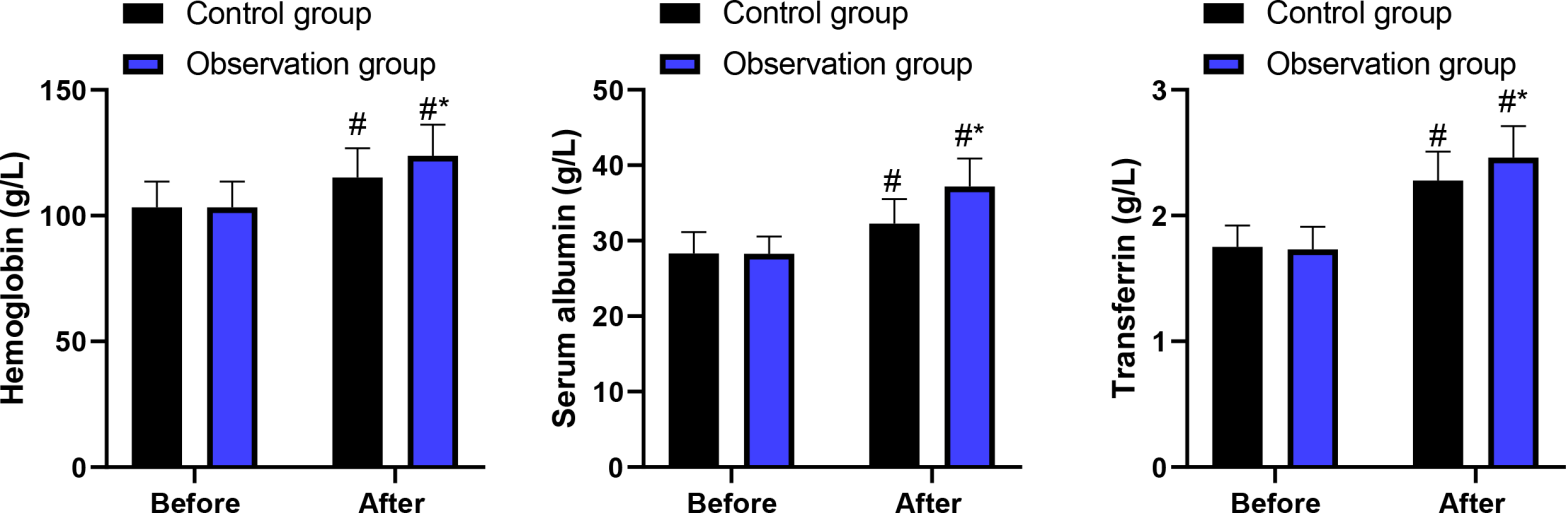
Hemoglobin, serum albumin, and transferrin levels in 2 groups. ^#^P<0.05, in contrast to before intervention. ^*^P<0.05, in contrast to control group.

Before intervention, no difference was discovered in hemoglobin, serum albumin, and transferrin levels between 2 groups (P > 0.05). After intervention, hemoglobin, serum albumin, as well as transferrin levels were increased in 2 groups, and those in the observation group presented higher when comparing with the control group (P < 0.05, **Figure 3**). After adjustment for baseline values in ANCOVA models, the observation group demonstrated significantly greater improvements in hemoglobin (F = 5.67, P = 0.020), serum albumin (F = 8.12, P = 0.006), and transferrin (F = 6.45, P = 0.013).

### Inflammatory markers in 2 groups

**Figure 4:**
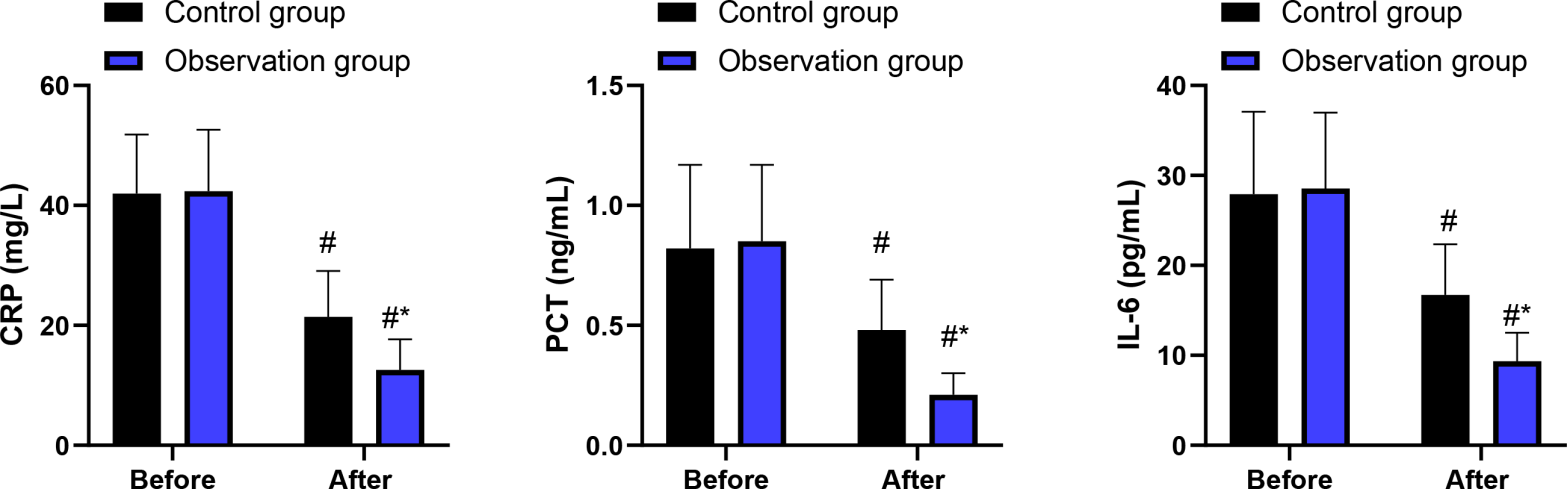
Levels of CRP, PCT, and IL-6 in 2 groups. ^#^P<0.05, in contrast to before intervention. ^*^P<0.05, in contrast to control group.

Prior to intervention, no significant differences were observed in CRP, PCT, or IL-6 levels between the two groups (P > 0.05). After intervention, levels of CRP, PCT, and IL-6 decreased significantly in both groups, with the observation group exhibiting significantly greater reductions compared with the control group (P < 0.05, **Figure 4**). ANCOVA analyses adjusting for baseline values confirmed that the observation group had significantly greater reductions in CRP (F = 7.45, P = 0.008), PCT (F = 6.89, P = 0.011), and IL-6 (F = 8.03, P = 0.006) compared to the control group.

### Immune function markers in 2 groups

**Figure 5:**
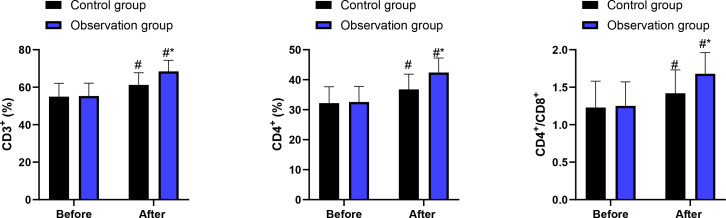
Percentages of CD3^+^ and CD4^+^, and the CD4^+^/CD8^+^ ratio in 2 groups. ^#^P<0.05, in contrast to before intervention. ^*^P<0.05, in contrast to control group.

Prior to intervention, no significant differences were reported between the two groups in percentages of CD3^+^ and CD4^+^, or the CD4^+^/CD8^+^ ratio (P > 0.05). After 2 weeks of intervention, the observation group exhibited significantly greater increases in CD3^+^, CD4^+^ percentages, and the CD4^+^/CD8^+^ ratio compared to the control group (P < 0.05, **Figure 5**). ANCOVA analyses, adjusting for baseline values, confirmed that the observation group had significantly greater improvements in CD3^+^ percentage (F = 6.54, P = 0.013), CD4^+^ percentage (F = 8.21, P = 0.005), and the CD4^+^/CD8^+^ ratio (F = 7.68, P = 0.007) compared to the control group.

### Quality of life in 2 groups

**Figure 6:**
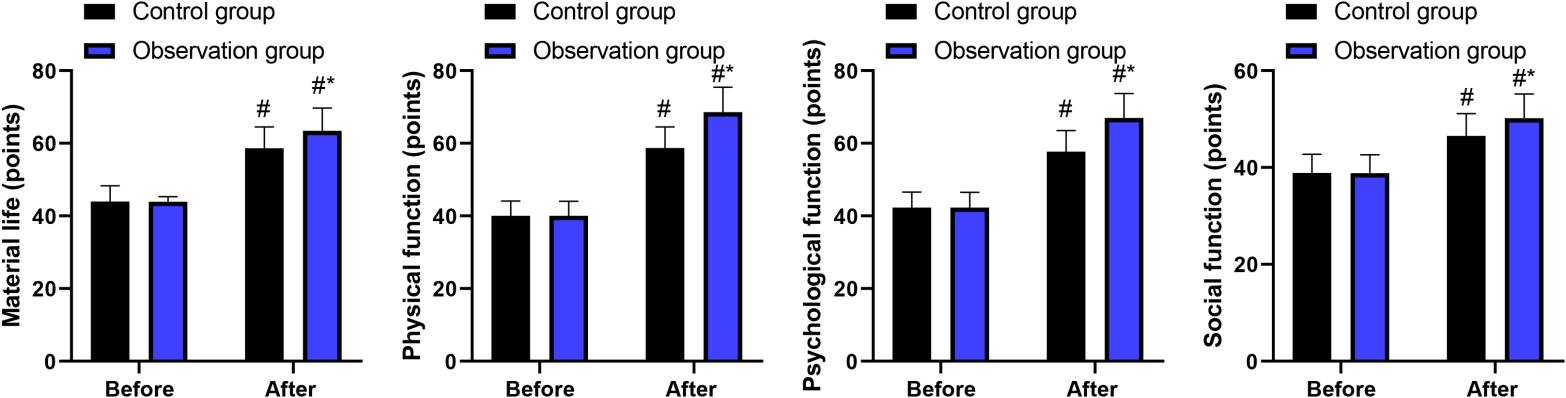
Quality of life in 2 groups. ^#^P<0.05, in contrast to before intervention. ^*^P<0.05, in contrast to control group.

Before intervention, no difference was discovered in the scores of material life, physical function, psychological function and social function between 2 groups (P > 0.05). After intervention, the scores of these aspects were elevated in 2 groups, and those in the observation group presented higher when comparing with the control group (P < 0.05, **Figure 6**). ANCOVA analyses adjusting for baseline scores confirmed significantly greater improvements in all four domains in the observation group (material life: F = 4.89, P = 0.030; physical function: F = 6.23, P = 0.015; psychological function: F = 5.78, P = 0.019; social function: F = 5.12, P = 0.026).

### Occurrence of adverse events in 2 groups

In contrast to the control group, the occurrence of adverse reactions in the observation group was lower (P < 0.05, **Table 3**). Multivariable logistic regression adjusting for potential confounders confirmed that the observation group had significantly lower odds of experiencing adverse events (adjusted OR = 0.35, 95% CI: 0.16-0.77, P = 0.009).

**Table 3:** Occurrence of adverse events in 2 groups.

Groups	n	Aspiration	Pulmonary infection	Upper gastrointestinal hemorrhage	Vomiting	Diarrhea	Abdominal distension	Total incidence rate
Control group	40	2	2	0	0	1	3	8 (20.00%)
Observation group	40	0	0	0	0	0	1	1 (2.50%)
χ^2^								6.135
P								0.013

### Need for alternative placement techniques

Need for alternative placement techniques was also assessed. In the control group, 10 patients (25.0%) required rescue placement under gastroscopic guidance after failed blind insertion, whereas in the observation group, only 2 patients (5.0%) required such rescue procedures (χ² = 6.275, P = 0.012).

## Discussion

The condition of patients undergoing severe thoracic surgery can change rapidly, and the gastrointestinal function of patients will be reduced rapidly after the onset, which will lead to nutritional deficiency in the body (9). However, in the state of malnutrition, the nutrients in the body of patients are difficult to meet their recovery needs, which will affect their recovery and prognosis (10). Therefore, during clinical treatment, the required nutrients of the patient’s body should be supplemented. However, there are differences in the use of different nutritional support methods, and different use of nutritional support methods will also lead to different treatment results (11). In the past, parenteral nutrition was mainly used in clinical treatment, which could meet the nutritional requirements of the body and improve the malnutrition status to a certain extent (12). However, patients with severe thoracic surgery were in the peak period of low metabolism 2 to 14 days after the onset of the disease, and it was difficult to meet the nutritional needs of patients with parenteral nutritional support alone (13). In the sequential enteral nutrition support mode, patients are first provided with short peptide ready-to-use type enteral nutrition preparations to promote patients to gradually adapt to the enteral nutrition support mode (14). After patients do not have complications such as vomiting, gastric bleeding, and reflux, whole protein enteral nutrition support preparations rich in various dietary fibers are gradually applied, so that the necessary nutritional support can be guaranteed (15). The use of sequential enteral nutrition support in the treatment of severe patients can make the body organs gradually adapt to avoid the situation of insufficient nitrogen and caloric intake in the early stage and nutrient deficiency in the later stage, so as to help enhance the body nutrition status of patients (16).

In recent years, sequential enteral nutrition support therapy has been widely used in clinical practice, but the previous blind nasoenteric tube insertion technology still has a low success rate and needs further improvement (17). As an important way of enteral nutrition support, indwelling nasoenteric tube can deliver pre-prepared nutrient solutions to the duodenum or jejunum of patients, which can help improve the absorption of protein, heat and other nutrients by the body (18). However, there are few clinical options for nasoenteric tube insertion, so scientific and accurate catheterization can effectively improve the efficiency of enteral nutrition absorption and improve the intervention effect (19). Some studies have shown that injecting water into the stomach when the nasoenteric tube is placed in indentation is conducive to identifying the arrival of the nasoenteric tube to the stomach, and at this time, ultrasound technology or X-ray can further identify the location of the nasoenteric tube, which is conducive to improving the pyloric passage rate of the tube (20).

The results of our study indicated that in contrast to the control group, the catheterization time and hospital stay in the observation group were lower. At the same time, the cases of catheterization success in the observation group were higher as comparing with the control group. These findings remained robust after multivariable adjustment, with the observation group demonstrating an approximately 3.4-fold higher odds of successful catheterization (adjusted OR = 3.42, 95% CI: 1.38-8.47) and significantly reduced catheterization time and hospital stay in adjusted analyses. It is noteworthy that the magnitude of differences observed in catheterization success and adverse events was relatively large. While these findings may reflect genuine procedural advantages, they also raise the possibility of bias. In particular, due to the inability to blind operators to group allocation, performance bias cannot be excluded. Operator experience, procedural familiarity, and subtle differences in technique execution may have contributed to the observed effect sizes. Therefore, these results should be interpreted with caution. Besides, after intervention, BMI, TSF and MAMC as well as hemoglobin, serum albumin, as well as transferrin levels were increased in 2 groups, and those in the observation group were higher when comparing with the control group. ANCOVA analyses confirmed that these improvements were independent of baseline values, suggesting a potential association after adjustment rather than a definitive causal treatment effect. All these findings suggested that the technique of blind naso-intestinal tube insertion by water injection, dilution and drainage method was associated with improved short-term nutritional status in this patient population.

Furthermore, the observation group showed significantly greater reductions in systemic inflammatory markers (CRP, PCT, IL-6) and significantly greater improvements in cellular immune function markers (CD3^+^, CD4^+^, CD4^+^/CD8^+^ ratio). This suggests that the combination of the enhanced catheterization technique and the sequential enteral nutrition protocol may be associated with improved outcomes beyond procedural success, although these downstream outcomes should not be interpreted as direct consequences of the catheterization technique itself. However, the higher catheterization success rate observed in the intervention group represents a procedural advantage, and extrapolating this advantage directly to systemic biological or clinical outcomes is not sufficiently justified. Once correct post-pyloric positioning is achieved, the insertion method itself is unlikely to independently determine inflammatory or immunological responses.

Several factors may contribute to these observed associations. It has been reported in the literature that the short peptide formula exists in the form of dipeptide and tripeptide, and its nutrient absorption does not require the coordination of digestive enzymes, and the fiber substance in the whole protein formula can promote gastrointestinal peristalsis and accelerate the recovery of gastrointestinal function (21). Additionally, the water injection, dilution and drainage technique involves operation steps that may be more convenient than conventional methods, potentially reducing the need for multiple gas injection auscultation procedures. The feedback information obtained from aspirated fluid may also provide more accurate guidance for tube positioning, with abdominal X-ray serving as the gold standard for confirmation (22). The reduced systemic inflammation and improved immune function observed are more likely secondary to improved nutritional delivery, earlier and more effective enteral feeding, and reduced complications, rather than a direct effect of the catheterization technique itself. However, it is important to emphasize that these proposed mechanisms—including stimulation of gastrointestinal peristalsis, enhanced nutrient absorption capacity, and improved catheterization accuracy—were not directly measured in the present study. The observed improvements in nutritional status and reduced hospitalization duration may result from a combination of factors related to both the catheterization technique and the sequential nutrition protocol, and further studies incorporating direct mechanistic measurements are needed to elucidate the underlying pathways.

Our study also indicated that after intervention, the scores of material life, physical function, psychological function and social function were elevated in 2 groups, and those in the observation group were higher compared with the control group. These findings were confirmed in ANCOVA analyses adjusting for baseline scores. Meanwhile, in contrast to the control group, the occurrence of adverse reactions in the observation group was lower, with multivariable logistic regression indicating a 65% reduction in the odds of adverse events (adjusted OR = 0.35, 95% CI: 0.16-0.77). These results suggest that blind naso-intestinal tube insertion technique using water injection, dilution and drainage method was linked to improved short-term quality of life and reduced adverse events in patients receiving enteral nutrition support.

Potential explanations for these associations may be considered based on previous research. It has been suggested that water injection techniques may facilitate observation of naso-intestinal tube passage when combined with abdominal X-ray, and that gastric water instillation might help contain nutrients within the small intestine, potentially reducing reflux and aspiration risk (23). Warm water stimulation has also been hypothesized to promote gastrointestinal motility and digestive function. The improvements in quality of life are likely a direct result of better nutritional status, fewer complications, and shorter hospital stays. However, these mechanistic pathways were not directly assessed in our study. The observed reduction in adverse events and improvement in quality of life likely reflect the combined benefits of more accurate tube placement and effective nutritional delivery, but the specific contributions of individual mechanisms remain speculative and require direct investigation in future studies. However, it should be noted that quality of life was assessed after only two weeks, which may not adequately reflect meaningful postoperative recovery. The observed changes are likely influenced by transient postoperative conditions, including acute physiological stress, pain, and hospitalization-related factors, and therefore should be interpreted with caution. Accordingly, the magnitude of the observed effects should be interpreted as preliminary, and confirmation in studies with more rigorous control of bias is required. In addition, given the relatively small sample size and the large number of outcomes assessed, there remains a potential risk of type I error. Therefore, findings related to secondary outcomes should be interpreted with caution and considered exploratory rather than confirmatory.

## Limitations

The limitations of this study should be considered. First, this study was conducted in a single, specialized thoracic surgery department, which may limit the generalizability of the findings. In addition, the intervention was highly protocol-dependent and performed by experienced operators under standardized conditions, which may not reflect routine practice in other institutions. Therefore, the applicability of these results to settings with different operator expertise, clinical workflows, or patient populations remains uncertain. Further multi-center studies involving larger and more varied patient groups are necessary to confirm our results.

Second, as noted in the Introduction, this study evaluated the combined clinical effectiveness of the entire intervention package, which included both the novel tube insertion technique and the sequential enteral nutrition strategy. Therefore, the observed improvements in nutritional outcomes and quality of life cannot be attributed solely to the catheterization method, as the sequential nutrition protocol itself may have contributed substantially to these effects. Importantly, these effects should be interpreted as arising from the combined procedural protocol rather than the catheterization technique alone. This represents a potential combined intervention bias, and future studies should consider factorial designs to isolate the independent contribution of each component.

Third, despite randomization, the two groups differed in several procedural elements beyond the primary insertion technique, including the administration route and timing of prokinetic agents, patient positioning, and workflow processes. These differences indicate that the present study evaluated a combined procedural intervention rather than an isolated comparison of catheterization techniques. Consequently, the observed effects cannot be attributed solely to the water injection, dilution and drainage method, and residual confounding cannot be excluded. Importantly, the distinction between procedural success and downstream clinical outcomes should be emphasized, as improvements in systemic parameters may reflect indirect effects rather than direct consequences of the intervention.

Fourth, although outcome assessors were blinded to group allocation, blinding of operators and patients was not feasible due to the nature of the interventions, which may introduce performance bias. Given the relatively large differences observed in catheterization success and adverse events, the potential impact of such bias should be carefully considered. These effect sizes may be influenced not only by the intervention itself but also by operator-related factors and procedural context. Additionally, the relatively short follow-up period (2 weeks) limits our understanding of the long-term effects of this technique on patient outcomes such as readmission rates or sustained nutritional improvements. The use of the GQOLI-74 at 2 weeks post-intervention may be subject to question regarding its temporal sensitivity in critically ill postoperative patients. While we observed improvements in all domains, the 2-week timeframe may be insufficient to capture meaningful changes in quality of life, and patients’ responses may be influenced by their acute postoperative state, pain, medication effects, or the hospital environment. Future studies should consider longer follow-up periods (e.g., 3–6 months) and the use of quality of life instruments specifically validated for critically ill or postoperative populations. Therefore, the quality-of-life findings in this study should be considered preliminary and may primarily reflect short-term postoperative conditions rather than sustained recovery.

Fifth, and importantly, the mechanistic interpretations discussed above-–including enhanced gastrointestinal motility, improved nutrient absorption, reduced aspiration, and modulation of inflammation and immunity-–were not directly measured in this study. These proposed mechanisms are speculative and based on physiological plausibility and previous literature rather than on direct evidence from our data. Future studies should incorporate direct measurements of gastrointestinal motility (e.g., gastric emptying studies, manometry), nutrient absorption markers, aspiration events, and specific inflammatory and immune pathways using objective methods to validate these proposed mechanisms.

Sixth, our study relied heavily on surrogate endpoints measured over a short 2-week follow-up period. Outcomes such as BMI, triceps skinfold thickness, mid-arm muscle circumference, serum albumin, transferrin, and CD4^+^/CD8^+^ ratio are indirect markers of nutritional and immune status. While they are standardized and commonly used in enteral nutrition research, they do not directly reflect patient-centered clinical outcomes such as aspiration pneumonia, feeding tolerance, long-term nutritional adequacy, or mortality. The low incidence of aspiration and pneumonia in both groups (2 events in controls, 0 in observation group) limited our ability to assess these hard endpoints meaningfully. Furthermore, we did not systematically measure feeding adequacy (e.g., percentage of prescribed calories delivered per day, time to reach target feeding rate), which is a key determinant of clinical benefit from enteral nutrition. The need for alternative placement techniques (gastroscopic guidance) was significantly lower in the observation group, but this outcome is still procedure-oriented rather than patient-centered. Future studies should prioritize clinically meaningful endpoints, include longer follow-up periods (e.g., 3–6 months), and systematically record feeding adequacy metrics.

Seventh, the study evaluated a relatively large number of outcomes in a modest sample size (n=80), which increases the risk of type I error despite the use of correction methods. Although the primary outcome was predefined, many secondary analyses should be considered exploratory. Future studies with larger sample sizes and pre-specified outcome hierarchies are needed to confirm these findings.

Eighth, feeding adequacy (e.g., the proportion of prescribed caloric delivery achieved) was not evaluated in this study. This represents an important limitation, as adequate delivery of enteral nutrition is a key determinant of clinical outcomes. Future studies should include quantitative assessments of nutritional delivery to better evaluate the clinical effectiveness of the intervention.

Despite these limitations, our study provides preliminary evidence that the water injection, dilution and drainage method may offer advantages over conventional gas insufflation for blind nasoenteric tube placement in patients undergoing sequential enteral nutrition. The additional improvements in inflammatory and immune markers suggest potential broader benefits that warrant further investigation. Given that the mechanistic pathways underlying these observations remain to be directly established, these findings should be interpreted with appropriate caution and considered hypothesis-generating rather than confirmatory. Well-designed, multi-center randomized controlled trials incorporating direct mechanistic measurements are required to confirm these findings and elucidate the underlying mechanisms.

## Conclusion

This study suggests that the combined procedural package for blind nasoenteric tube insertion is associated with improved catheterization success and procedural efficiency compared with a conventional approach. However, given the presence of multiple co-interventions, the lack of intervention isolation, and the short follow-up period, these findings should be interpreted with caution. The observed improvements in nutritional status, inflammatory markers, immune function, and quality of life are likely indirect and should not be attributed to the catheterization technique itself. Overall, the results primarily support a procedural advantage rather than a direct clinical or biological effect. These findings should be considered exploratory and hypothesis-generating, and require confirmation in larger, multicenter studies with clearer intervention separation and clinically meaningful endpoints.

## Data Availability

The original contributions presented in the study are included in the article/**Supplementary Material**. Further inquiries can be directed to the corresponding author.

## Supplementary material

Supplementary Figure 1 Schematic illustration of the key steps in the water injection, dilution and drainage method for blind nasoenteric tube placement, including gastric water instillation, tube advancement, aspiration and pH testing, and final positioning confirmed by X-ray.
