# Supplementary figures and images for: Clinical value of the water injection, dilution, and drainage method for blind nasoenteric tube placement in patients receiving sequential enteral nutrition support after major thoracic surgery
Source: Front Oncol. 2026 May 19;16:1838370. doi: 10.3389/fonc.2026.1838370 (PMC13226009; doi:10.3389/fonc.2026.1838370)

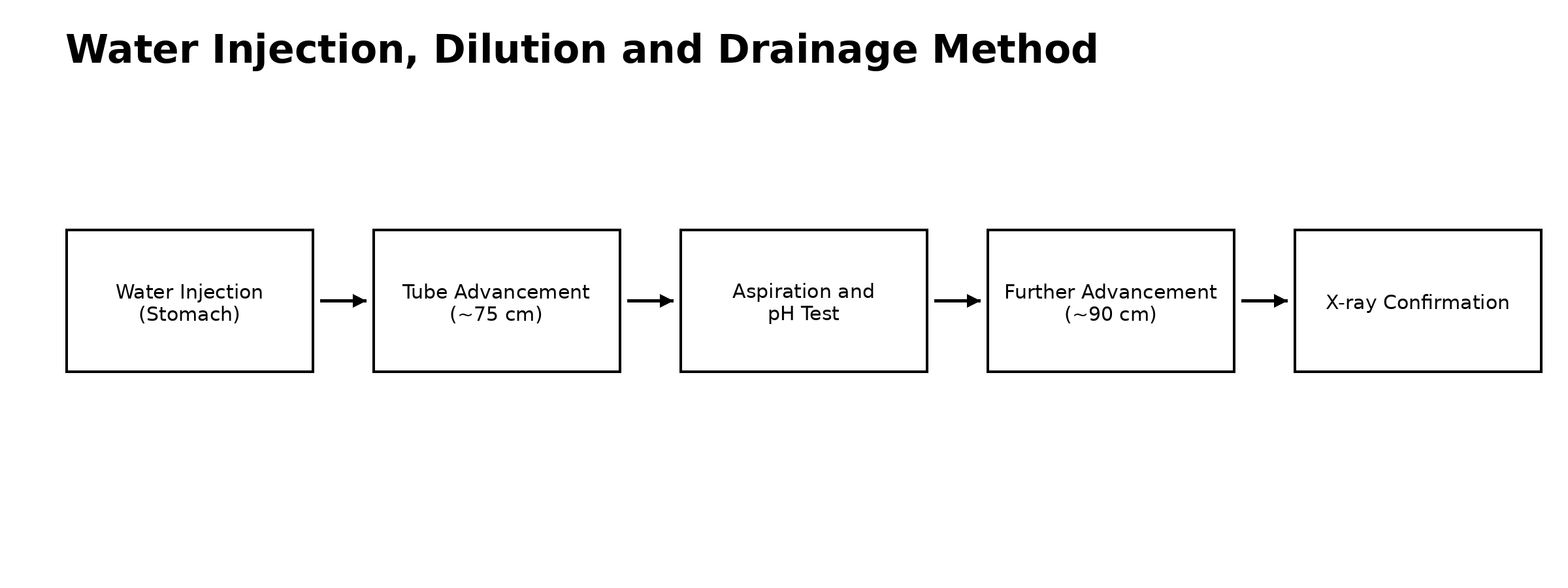

Supplement: Supplementary Figure 1 — Schematic illustration of the key steps in the water injection, dilution and drainage method for blind nasoenteric tube placement, including gastric water instillation, tube advancement, aspiration and pH testing, and final positioning confirmed by X-ray. [file Image1.tiff]
